# Reimagining maternal and infant wellbeing through AI-embedded integrative arts-based care: a public health perspective

**DOI:** 10.3389/fpubh.2026.1899694

**Published:** 2026-08-17

**Authors:** Feng Li, Sinuo Chen, Haoyu Suo, Chengmeng Zhang

**Affiliations:** 1 School of Music, Anyang Normal University, Anyang, Henan Province, China; 2 Art & Technology/Sound Practices Department, School of the Art Institute of Chicago (SAIC), Chicago, IL, United States; 3 Social Development Research Institute, National Development and Reform Commission of China, Beijing, China; 4 Institute of Population Research, Peking University, Beijing, China; 5 International Research Institute of Ageing, Hong Kong, Hong Kong SAR, China

**Keywords:** artificial intelligence, arts-based care, maternal and infant health, non-pharmacological interventions, perinatal care, public health

## Abstract

The peripartum period and early parenting are critical phases for maternal health, infant development, family functioning, and long-term wellbeing. Although maternal and child health services have traditionally prioritized pregnancy safety, childbirth security, and neonatal survival, contemporary public health agendas increasingly emphasize maternal mental health, positive birth and postnatal experiences, parent-infant bonding, and family-centered support. Evidence suggests that music therapy, mindful music listening, artistic expression, doula support, aromatherapy, parent-infant singing, and parental voice interaction may help reduce pregnancy related anxiety, labor pain, fear of cesarean birth, postpartum psychological distress, and neonatal pain, while also supporting early relational care. However, current applications remain fragmented, often limited to single interventions, isolated settings, short care periods, and insufficient personalization. This Perspective proposes an AI-embedded integrative arts-based care framework for promoting maternal and infant wellbeing across pregnancy, childbirth and cesarean birth, postpartum recovery, neonatal intensive care, and home and community follow up. Within this framework, artificial intelligence is conceptualized as a supportive coordination infrastructure rather than a substitute for professional care. It may assist risk identification, needs profiling, personalized intervention matching, dynamic monitoring, adaptive feedback, referral linkage, and continuity across hospital, community, and family settings. The framework further emphasizes professional oversight, human verification, privacy protection, equitable access, and ethical governance. By integrating arts-based support with responsible AI-enabled coordination, this Perspective reimagines non-pharmacological maternal and infant care as a continuous, human-led, family-participatory public health pathway for diverse perinatal care contexts.

## 1 Introduction

Maternal and infant health has long been a central concern of public health systems. Conventional perinatal services have been organized primarily around maternal safety, safe childbirth, prevention of obstetric complications, reduction of neonatal mortality, and the management of immediate clinical risks (1). These objectives remain indispensable. At the same time, pregnancy, labor, cesarean birth, postpartum recovery, neonatal care, and early parenting are emotionally intense, socially embedded, relationally significant, and developmentally formative experiences (2). Women may encounter anxiety, fear of childbirth, pain, loss of control, sleep disruption, role transition, breastfeeding pressure, concerns about infant care, and changing family relationships. Families may also experience uncertainty, caregiving burden, and difficulties in building early parent-infant relationships. Maternal and infant health should therefore be understood not only as the absence of mortality, morbidity, or clinical complications, but also as the presence of dignity, emotional security, supportive relationships, parental confidence, positive childbirth and postnatal experiences, and early developmental support.

This broader understanding is increasingly reflected in international maternal and newborn care guidance. The World Health Organization has emphasized positive childbirth experience and positive postnatal experience including respect, dignity, emotional support, informed decision making, family participation, effective communication, and the physical and psychosocial wellbeing of women and newborns (3, 4). It is also important to avoid treating current care systems as uniform. Care models differ substantially across countries, institutions, and clinical settings. In some contexts, family-centered and neurodevelopmentally supportive care has been established for decades, particularly in neonatal care traditions (5).

Supportive approaches such as music therapy, music listening, artistic expression, doula-support care, parent-infant singing, and other sensory or relational interventions may respond to aspects of perinatal and neonatal care, that cannot be fully addressed by medication or procedural intervention alone. They should not be understood merely as optional comfort measures. Rather, they may contribute to a broader public health pathway for improving maternal and infant wellbeing across the perinatal continuum. These approaches may help women and families organize difficult emotions, reduce physiological arousal, reshape the perception of pain, create a more supportive birth environment, strengthen parental agency, and support early relational experiences between parents and infants (6–8). In pregnancy, music and arts-based expression may offer a low threshold way to respond to anxiety, uncertainty, and fear of childbirth. During labor and cesarean delivery, music, breathing, rhythm, and continuous support may contribute to a greater sense of control and emotional safety (9–11). In the postnatal and NICU settings, parental voice, singing, and gentle sensory interaction may help transform parents from passive observers into active participants in early care, support emotional expression, parent–infant interaction, and family participation.

Nevertheless, these forms of support are often studied or delivered as isolated interventions, local innovations, or short-term programmes. Their connection with screening, referral, professional supervision, family participation, and hospital–community–home continuity remains limited (12). The main question is therefore not only whether a single arts-based intervention can reduce a single symptom, but how different forms of arts-based support can relate to routine maternal care, neonatal care, community follow up, and family based early parenting support. On this basis, this Perspective proposes an AI-embedded integrative arts-based care framework for promoting maternal and infant wellbeing across pregnancy, childbirth, the postnatal period, neonatal care, and early family life.

## 2 Evidence base and integration gaps in arts-based care and related supportive practices

### 2.1 Conceptual classification of supportive practices

The interventions discussed in this Perspective should not be treated as a single homogeneous category. Arts and health literature emphasizes the need to distinguish among arts modalities, forms of engagement, mechanisms of action, delivery contexts, and professional requirements (13, 14). Even within live music interventions in pediatric hospital settings, substantial heterogeneity exists in aims, participants, delivery methods, provider roles, and reported outcomes (12). This distinction is important for the present framework because maternal and infant care involves expressive, sensory, relational, and professionally mediated forms of support.

For conceptual clarity, this article distinguishes among three related forms of support. The first consists of core arts-based modalities, including music therapy, music listening, artistic expression, parent–infant singing, and parental voice interaction (15, 16). The second consists of arts-adjacent sensory and embodied supports, such as rhythm and breathing, environmental sensory adjustment, and aromatherapy. These are not all arts interventions in a strict sense, but they may shape the sensory and embodied conditions through which arts-based care is experienced (17). The third consists of relational and professional enabling practices, such as doula support, kangaroo care, family-centered care, and therapist-mediated parental participation (18, 19). These practices are not arts modalities themselves, but they provide the relational, professional, and contextual conditions through which arts-based care can be safely and meaningfully delivered.

In this article, “integrative arts-based care” therefore refers not to the simple aggregation of heterogeneous interventions, but to a care pathway in which core arts-based modalities are coordinated with sensory, relational, and professional supports according to clinical context, family needs, and professional judgment.

### 2.2 Pregnancy: emotional support and meaningful maternal experience

Anxiety, stress, fear of childbirth, and depressive symptoms during pregnancy may affect women's subjective wellbeing, sleep quality, participation in prenatal care, family relationships, childbirth preparation, and postpartum adjustment. Existing systematic reviews and meta-analyses suggest that music interventions during pregnancy may help reduce maternal anxiety and support stress relief and psychological wellbeing, although effects vary by music selection, intervention frequency, risk level, and implementation context (9, 20, 21). Arts-based interventions have also shown potential for improving psychological symptoms during pregnancy and the postpartum period, but the evidence remains heterogeneous (11). Within the taxonomy proposed above, pregnancy-related support mainly involves core arts-based modalities, such as music listening and artistic expression, together with low-intensity sensory or embodied practices such as rhythm, breathing, and guided relaxation.

The main gap is not the absence of supportive modalities, but the weak connection between these modalities and routine perinatal services. Many prenatal interventions are short-term and single component, with limited linkage to antenatal screening, prenatal education, obstetric outpatient services, birth planning, partner involvement, or postpartum follow-up. Consequently, prenatal arts-based care and related supportive practices often remain temporary emotional relief activities rather than part of a continuous psychological support pathway.

### 2.3 Labor and cesarean birth: pain coping, agency, and environmental support

Labor pain is shaped not only by physiological processes, but also by fear, attention, communication, perceived control, environmental safety, cultural expectations, and support from caregivers and companions. Existing reviews suggest that music-based interventions may reduce pain and anxiety during labor and cesarean delivery, although the quality and consistency of evidence remain limited and intervention protocols differ substantially (7, 22). Continuous doula support has also been associated with improved childbirth experiences and selected clinical outcomes (23). In this setting, music and rhythm-based support may be understood as core or arts-adjacent modalities, whereas doula support functions as a relational enabling practice that shapes communication, emotional security, and the care environment.

Cesarean birth further demonstrates the need for preference-based and context-sensitive support. Evidence that standardized Mozart music does not consistently improve all satisfaction or anxiety outcomes during cesarean delivery indicates that the effectiveness of music support cannot be separated from individual preference, surgical setting, clinical procedure, and implementation method (24). The major gap is that labor and cesarean supportive practices are often treated as single-point pain or anxiety interventions, with insufficient continuity from prenatal preparation to intrapartum care, postoperative reflection, and postpartum emotional recovery.

### 2.4 Postpartum and NICU care: emotional expression, infant comfort, and family participation

The postpartum period involves physical recovery, sleep deprivation, breastfeeding pressure, role transition, parenting fatigue, and reorganization of family relationships. Perinatal mental health services need to identify depression, anxiety, bipolar disorder, self-harm risk, and postpartum psychosis and ensure timely treatment and referral (25–27). Existing studies suggest that arts-based interventions may provide non-stigmatizing forms of emotional expression and social connection during pregnancy and the postpartum period (11). Digital health interventions have also shown low-to-moderate effects on perinatal depressive symptoms, providing indirect support for future technology-assisted screening and follow-up models (28).

In neonatal intensive care, music and parental voice interaction have been used as non-pharmacological approaches for supporting infant comfort, reducing procedural pain or stress responses, and strengthening family participation. Existing studies suggest that music may alleviate pain and stress responses during routine procedures in preterm infants; maternal singing and speaking may support autonomic nervous system maturation; and parent-led infant-directed singing appears safe and acceptable, although long-term parent–infant bonding effects remain mixed (6, 10, 29). Singing in the NICU is not simply an auditory stimulus or a parent-delivered task. It may be experienced as a relational practice that supports parental agency, emotional closeness, and parent–infant interaction when embedded in family-centered care and professional guidance (18). Neonatal pain management also increasingly emphasizes multidimensional and family-centered approaches (30).

The integration gap is clear. Postpartum mental health support is often weakly connected with clinical screening, community visits, and referral pathways. NICU voice- and music-based practices are frequently limited to hospitalization and insufficiently linked to post-discharge family support, community follow-up, or continuing parental confidence-building. Fathers, grandparents, and other caregivers are underrepresented. Postpartum mental health, infant comfort, parent–infant bonding, parental self-efficacy, and community support are rarely evaluated within a unified framework.

### 2.5 From singular evidence to continuous integration

Overall, the evidence base supports cautious development rather than overclaiming. Current evidence suggests that selected arts-based and non-pharmacological interventions may support emotional regulation during pregnancy, pain and anxiety coping during labor and cesarean birth, postpartum emotional expression, and neonatal comfort or family participation. However, these findings are distributed across different settings and disciplinary boundaries and have not yet developed into a continuous support system for maternal and infant wellbeing.

Four integration gaps are especially important. First, there is insufficient conceptual differentiation across modalities and enabling practices: music therapy, music listening, artistic expression, aromatherapy, doula support, kangaroo care, parent–infant singing, and parental voice interaction are not equivalent interventions and require different professional, relational, and contextual conditions. Second, there is insufficient continuity across stages: pregnancy, labor, cesarean care, postpartum recovery, NICU care, and home follow-up are treated as disconnected periods. Third, there is insufficient personalization by risk level, preference, culture, family resources, and digital access. Fourth, there is insufficient system embedding in maternal and child healthcare, community follow-up, referral mechanisms, and professional workflows. The proposed AI-embedded framework responds to these gaps, but it should be understood as a conceptual extension requiring empirical validation (Table 1).

**Table 1:** Existing evidence and major integration gaps in arts-based care for maternal and infant health.

Application area	Main modalities or supportive practices	Current evidence base	Main integration gaps
Prenatal emotional support	Music listening, music-based relaxation, art expression, breathing and rhythm training	Music interventions may reduce anxiety, stress, and some depressive symptoms during pregnancy; arts-based interventions may support perinatal mental health [Lin et al. (9); Maul et al. (21); Qian et al. (11); Shafqat et al., (31)].	High heterogeneity, limited personalization, insufficient linkage with antenatal education and postpartum follow-up.
Labor pain and anxiety	Music, doula support, aromatherapy, breathing rhythm, partner-supported sensory care	Music may support non-pharmacological management of labor pain and anxiety; continuous doula support improves childbirth experiences and selected outcomes [Bohren et al. (23); Hunter et al. (7); Santiváñez-Acosta et al. (22)].	Mostly single-point interventions; weak real-time feedback, limited multimodal integration, and inadequate connection with prenatal preparation.
Cesarean birth experience	Individualized music, preoperative relaxation, intraoperative auditory support, postoperative reflection	Evidence is inconsistent; standardized music does not necessarily improve all satisfaction or anxiety outcomes, indicating the need for contextual and preference-based adaptation [Drzymalski et al. (24)].	Insufficient perioperative continuity, limited preference assessment, and weak integration with surgical communication and postoperative emotional recovery.
Postpartum mental health	Music relaxation, art expression, group arts activities, mother-infant singing, narrative or visual expression	Arts-based interventions may provide supportive effects for postpartum anxiety and depressive symptoms; digital health interventions show low-to-moderate effects on perinatal depressive symptoms [Anyanwu & Jenkins (28); Qian et al. (11); Shen et al., (32)].	Limited linkage with mental-health screening, referral pathways, community follow-up, and family support systems.
Neonatal/NICU care	Parental voice, maternal humming or singing, parent-led infant-directed singing, music soothing	Music and parental voice may alleviate procedural pain and stress in preterm infants; parent-led singing is safe and acceptable, although long-term bonding effects remain mixed [Filippa et al. (29); Ghetti et al. (6); Ou et al. (10)].	Unclear safety dosage, sound intensity, infant-state adaptation, professional supervision, and post-discharge continuity.
Hospital-community-family continuity	Digital platforms, remote support, family interaction tasks, follow-up prompts	AI and digital tools may assist screening, risk prediction, follow-up, clinical decision support, and information linkage across care settings [Kwok et al. (33); Lin et al. (33)].	Weak system embedding, privacy and governance risks, digital divide, and unclear accountability across institutions.

## 3 An AI-embedded integrative arts-based care framework

### 3.1 Conceptual boundary and overall framework

The framework proposed in this Perspective should be understood as a conceptual extension of the current evidence base, rather than as a clinically validated intervention model. Existing evidence mainly concerns selected arts-based and non-pharmacological interventions in specific perinatal or neonatal settings, including music listening, music therapy, artistic expression, doula support, parent–infant singing, and parental voice interaction (6, 7, 20, 23, 24, 29). These interventions have been examined in relation to prenatal anxiety and psychological wellbeing, labor pain and anxiety, cesarean birth experience, postpartum emotional distress, neonatal comfort, and parent–infant bonding (9–11, 21, 22). These studies provide the empirical basis for selected components of the framework, but they should not be read as direct evidence for the effectiveness of the proposed integrated AI-embedded pathway.

This distinction is central to the argument of this article. The AI-embedded framework proposed here concerns how such arts-based care within maternal and infant health could be coordinated, personalized, monitored, and linked across settings. AI is therefore not presented as an evidence-based treatment. Rather, it is conceptualized as a supportive coordination infrastructure that may, in principle, assist with risk identification, needs profiling, personalized matching, dynamic monitoring, adaptive feedback, referral and linkage, and continuity of support. Related work on digital health interventions, machine learning, and AI-supported maternal health suggests possible roles for screening, risk prediction, follow-up, clinical decision support, and information linkage, but these functions remain indirect evidence for the present framework and require specific validation in AI-embedded arts-based care (28, 33, 34).

In this framework, “integrative” has three meanings. First, it refers to integration across care settings, including prenatal clinics, prenatal education, labor and delivery units, cesarean care, postpartum wards, NICUs, community follow-up, and home-based parenting. Second, it refers to integration across arts-based modalities and related sensory or relational supports, including music listening, rhythm and breathing, artistic expression, sensory support, doula-supported care, and parent–infant voice interaction. Third, it refers to integration across stakeholders, including obstetricians, midwives, nurses, mental health professionals, music therapists, art therapists, doulas, community health workers, mothers, fathers, and other family caregivers. The aim is not to add more artistic activities to every stage of care, but to build a more coherent pathway through which appropriate low-risk supportive resources could be connected with professional assessment, family participation, and community continuity.

**Figure 1:**
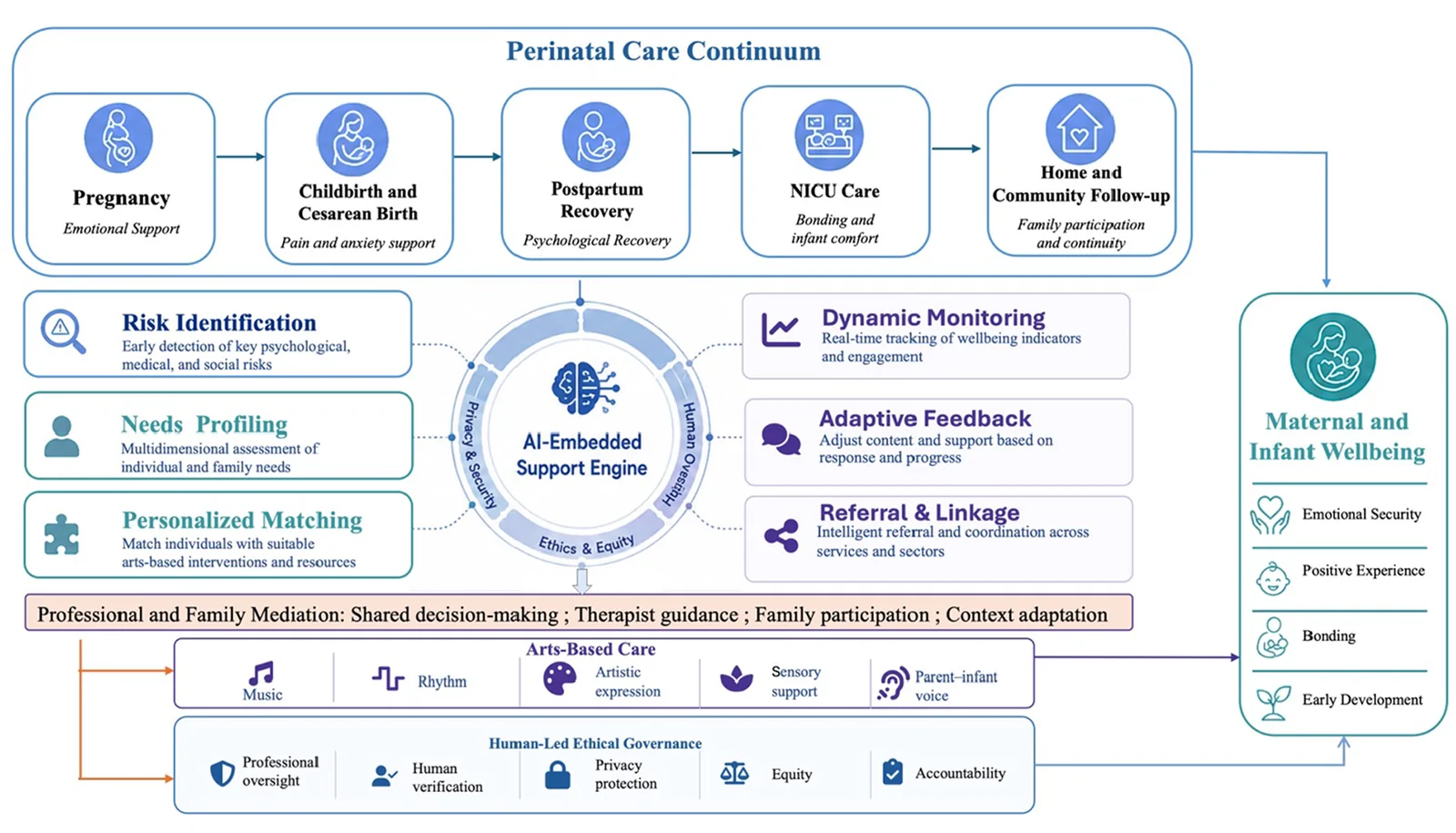
AI-embedded integrative arts-based care framework for maternal and infant wellbeing.

Figure 1 presents the conceptual framework of AI-embedded integrative arts-based care for maternal and infant wellbeing. The figure places the AI-embedded support engine at the center of the framework, linking the perinatal care continuum, arts-based care modalities, human-led governance, and maternal and infant wellbeing outcomes. It also emphasizes that AI-supported functions should operate under privacy protection, ethics and equity, and human oversight.

As shown in Figure 1, the proposed framework does not position AI as an autonomous therapeutic agent. More broadly, this framework is also informed by the idea of socially embedded AI, which emphasizes that AI systems should be situated within human relationships, care contexts, and professional or family support networks rather than functioning as detached technical substitutes, because maternal and infant wellbeing also depends on relational care, family participation, and trusted human mediation (35). AI should not independently diagnose perinatal depression, anxiety disorders, self-harm risk, postpartum psychosis, obstetric complications, neonatal abnormalities, or parent–infant relational difficulties. It should also not replace clinical communication, emotional support, or professional referral. Its potential value lies in helping care teams recognize needs earlier, coordinate low-risk supportive resources more efficiently, and maintain continuity across hospital, community, and family settings.

### 3.2 Operational pathway in clinical practice

The proposed framework would require a cautious, staged, and human-supervised workflow. It does not assume that all coordination functions require AI. Many aspects of supportive maternal and infant care can and should be achieved through interdisciplinary collaboration, clinical pathways, community follow-up, and conventional digital health systems. The added value of AI is more limited: it may be most useful when care teams need to interpret multi-source and longitudinal information, prioritize follow-up across large caseloads, identify changes in risk or engagement over time, and link families with appropriate low-risk resources or referral pathways across hospital, community, and home settings. While from a technical perspective, the AI-supported layer could combine structured and unstructured information without converting such information into autonomous clinical decisions. Structured inputs may include electronic health record variables, obstetric history, NICU care records, validated screening scores, follow-up attendance, and self-reported symptom scales. Unstructured or qualitative inputs may include brief clinical notes, family preferences, music preferences, reported childbirth concerns, parent–infant interaction notes, and free-text follow-up responses. Natural language processing could be used to organize these qualitative inputs, identify recurring concerns, and support documentation, then multimodal predictive or rule-assisted algorithms could combine structured screening results, longitudinal self-reports, clinical context, and care preferences to support risk stratification, follow-up prioritization, and resource matching (36). Because perinatal and neonatal conditions may change rapidly, the system should also be designed to handle non-stationary longitudinal data cautiously. Rather than generating alerts from single minor fluctuations, the AI-supported layer should use clinically defined thresholds, time-windowed trends, repeated signal confirmation, and tiered alert levels. For example, mild and transient changes in sleep, anxiety, or engagement may trigger non-urgent follow-up prompts, whereas repeated deterioration or critical screening responses should trigger professional review (37). This design can reduce excessive false-positive alerts while ensuring that clinically important changes are not missed. Model performance, alert frequency, false-positive burden, and subgroup performance should also be periodically audited before any wider implementation. AI is therefore framed as a decision-support layer for pattern recognition, adaptive prioritization, and continuity, rather than as a provider of care, therapeutic presence, shared decision-making, or clinical judgment. Operationally, four components are especially important: data inputs, AI-assisted functions, clinical workflow, and professional oversight.

First, data inputs should be clearly defined and minimized. Relevant clinical information may include routine electronic health record data, pregnancy and obstetric history, prenatal visit information, labor and cesarean records, NICU care records, postpartum follow-up data, and results from validated screening tools for anxiety, depression, fear of childbirth, pain, sleep, parenting stress, and perceived social support. Existing guidelines emphasize the importance of screening, diagnosis, treatment, and referral for perinatal mental health conditions, particularly depression, anxiety, bipolar disorder, self-harm risk, and postpartum psychosis (25–27). Non-clinical information may also be relevant, including preferred music style, language, cultural background, availability of family support, digital access, and willingness to participate in arts-based activities. Sensitive materials such as parental voice recordings, infant images, parent–infant interaction records, or artistic expression products should be collected only when necessary, with explicit consent, clear retention rules, and options for withdrawal.

Second, AI-assisted functions should remain limited to coordination and decision support. These functions may include risk stratification, needs profiling, matching families with suitable low-risk arts-based resources, generating follow-up reminders, flagging changes in self-reported wellbeing, and suggesting referral pathways. Research on AI and machine learning in healthcare has highlighted the potential of data-driven systems for risk identification, high-risk patient management, and clinical decision support, while also emphasizing that clinical usefulness depends on data quality, workflow integration, interpretability, and accountability (38–40). In the present framework, such functions should be applied only as supportive tools. For example, a low-risk pregnant woman reporting mild anxiety may be offered optional music-based relaxation or breathing resources through a prenatal education program. By contrast, when validated screening scores, explicit self-harm statements, postpartum psychotic symptoms, acute obstetric concerns, serious neonatal deterioration, or other predefined high-risk signals are detected, a fail-safe escalation rule should override routine personalization. In such cases, the system should suspend low-risk arts-based recommendations for that episode, generate an urgent alert for professional review, direct the family to appropriate clinical or emergency contact pathways according to local protocols, record the escalation, and require human acknowledgment. This fail-safe function is intended to prevent supportive digital tools from creating a false sense of security or delaying urgent medical assessment.

Third, the clinical workflow should be risk stratified. Low-risk families may receive general arts-based care resources, such as music listening, rhythm and breathing, pregnancy journaling, parent–infant singing, or community group activities. Moderate-risk families may require structured follow-up by midwives, nurses, community health workers, or trained therapists. High-risk families, including those experiencing self-harm risk, postpartum psychosis, severe anxiety or depression, obstetric complications, preterm infants with complex care needs, or inadequate family support, should be referred to appropriate clinical services. In this workflow, AI may organize information and prompt action, but it should not determine the level of care without human review.

Fourth, professional oversight is essential. AI-generated outputs should be reviewable, explainable, and traceable. Care teams should be able to see what information informed a recommendation, whether the recommendation was accepted or rejected, and who made the final decision. Ethical discussions of machine learning in health care have repeatedly emphasized risks related to bias, opacity, accountability, and inappropriate reliance on automated outputs (41, 42). Therefore, professionals should retain responsibility for clinical interpretation, risk assessment, referral, and communication with families. Families should also be informed that AI-assisted recommendations are supportive rather than diagnostic or mandatory.

### 3.3 Risk-stratified application across the perinatal continuum

Across pregnancy, AI-embedded arts-based care could support early recognition of anxiety, fear of childbirth, sleep problems, or limited social support, and could connect women with appropriate low-intensity resources such as music listening, rhythm and breathing exercises, artistic expression, or partner-supported activities. These applications should be linked to prenatal education, routine screening, and referral pathways. They should not be delivered as isolated digital tasks. Existing evidence on music and arts-based interventions during pregnancy suggests potential benefits for anxiety, stress relief, and psychological wellbeing, but also indicates heterogeneity in intervention design, population, setting, and outcome measurement (9, 11, 20, 21).

During labor and cesarean birth, the framework should be more cautious because clinical safety and workflow constraints are more immediate. AI-assisted tools may be useful before delivery for documenting music preferences, childbirth-related fears, coping preferences, communication needs, and partner involvement. During labor or surgery, however, decisions about music, breathing, sensory support, or environmental adjustment should remain under the supervision of midwives, nurses, anesthesiologists, obstetricians, doulas, or therapists. Evidence on music-based interventions for labor and cesarean delivery suggests possible effects on pain and anxiety, while also showing limitations in evidence quality and intervention consistency (7, 22). Continuous doula support has also been associated with improved childbirth experiences and selected clinical outcomes (23). The inconsistent findings regarding standardized music during cesarean delivery further suggest that preference, setting, procedure, and implementation method must be considered (24).

In the postpartum period, AI-supported coordination could help connect mood screening, sleep reports, breastfeeding stress, parenting stress, family support, and arts-based resources. Music listening, artistic expression, mother–infant singing, and group-based arts activities may offer non-stigmatizing forms of emotional expression and social connection. Existing research suggests that arts-based interventions may provide psychological support during pregnancy and the postpartum period, while digital health interventions show low-to-moderate effects on perinatal depressive symptoms (11, 28, 43). However, persistent low mood, severe anxiety, self-harm risk, bipolar disorder, or postpartum psychosis requires professional assessment and treatment rather than reliance on digital arts-based support (25–27).

In NICU and post-discharge settings, the framework may support carefully supervised parent–infant voice interaction, parental singing, and family participation. Music and parental voice interaction have been used to support infant comfort and reduce procedural pain or stress responses, and parent-led infant-directed singing appears safe and acceptable, although long-term bonding effects remain mixed (6, 10, 29). Neonatal pain management also increasingly emphasizes multidimensional and family-centered approaches (30). Because preterm and critically ill infants are sensitive to sound intensity, stimulation frequency, and clinical state, any music or voice-based activity should be guided by NICU professionals and adapted to infant stability, care schedule, and parental readiness. After discharge, low-risk voice interaction and parent–infant singing may be continued as part of family support, while parental distress or low caregiving confidence should trigger community follow-up or referral. The framework should therefore be understood as relationally mediated. Many arts-based and music-based practices do not work as detachable “content” that can simply be selected by an algorithm. Their meaning and effects depend on who delivers them, how they are introduced, whether families accept them, and whether they are embedded in a trustworthy care relationship. Parental singing in neonatal care may support parental agency, parent–infant interaction, and emotional closeness when it is embedded in kangaroo care and supported by professional guidance (18). In such contexts, AI-supported personalization should not prescribe the intervention. It should help professionals and families identify possible supportive options, document preferences, and maintain continuity, while the actual decision and delivery remain grounded in family-centered care, professional competence, shared decision-making, and contextual adaptation.

Overall, the framework is intended to support continuity without turning maternal and infant care into an automated digital system. Across all stages of the perinatal continuum, high-risk signals should override routine personalization. If severe maternal mental health symptoms, active self-harm risk, postpartum psychosis, acute obstetric concerns, neonatal instability, or serious family safety concerns are identified, the framework should shift from supportive resource matching to urgent professional escalation. This rule is especially important in postpartum, NICU, and home follow-up settings, where digital continuity tools may otherwise mask rapid deterioration or delay direct professional contact. The practical value of this framework depends on whether it can strengthen professional coordination, preserve human relationships, reduce fragmentation, and expand equitable access to low-risk supportive resources across hospital, community, and family settings.

## 4 Risks, governance, and implementation challenges

### 4.1 Clinical safety, explainability, and professional accountability

Although the proposed framework may offer a pathway for integrating arts-based care into maternal and infant health services, its implementation raises clinical, ethical, legal, and organizational challenges. The proposed framework must be implemented with clear clinical boundaries (44). AI-assisted systems may support screening reminders, risk alerts, resource matching, and referral coordination, but they should not independently diagnose, treat, or manage perinatal mental disorders, obstetric risks, neonatal abnormalities, or family crises (45). Conditions such as self-harm risk, postpartum psychosis, severe depression, severe anxiety, bipolar disorder, obstetric complications, and complex neonatal conditions require professional assessment and clinical management.

Explainability is especially important in maternal and infant care. If an AI system assigns a risk level, suggests a supportive activity, or recommends referral, professionals should be able to understand the basis of that output. Black-box recommendations may create false reassurance, unnecessary anxiety, or unclear responsibility. Therefore, AI-generated recommendations should be traceable, reviewable, and linked to clearly defined clinical pathways. The system should record the data used, the recommendation generated, the professional who reviewed it, and the final action taken.

Legal and professional responsibility must also be clarified before implementation. When AI-generated recommendations are inaccurate, delayed, biased, or ignored, responsibility may become unclear among platform providers, healthcare institutions, clinicians, community workers, and families. To avoid this problem, AI should be positioned as a decision-support tool rather than an autonomous care provider. Healthcare professionals and care teams should retain responsibility for interpretation, communication, referral, and clinical decision-making.

### 4.2 Data protection, cybersecurity, and algorithmic bias

Maternal and infant health data are highly sensitive. The proposed framework may involve information on pregnancy, childbirth experience, mental health, sleep, pain, family relationships, infant health, parental voice, parent–infant interaction, and materials generated through artistic expression (46). Such information may reveal psychological distress, family conflict, social vulnerability, or infant health risks (47). Data collection should therefore follow the principles of necessity, proportionality, minimization, informed consent, and withdrawal.

Cybersecurity is not a secondary technical issue. If maternal mental health records, infant health data, voice recordings, or family interaction data are leaked, misused, or commercially exploited, families may experience stigma, discrimination, emotional harm, or loss of trust in healthcare services (46, 47). Systems should adopt secure storage, access control, encryption, audit trails, and clear rules for data retention and deletion. Sensitive data such as parental voice recordings, infant images, and mental health screening results should receive higher levels of protection.

Algorithmic bias may also reproduce or amplify existing inequalities. AI systems trained on data from urban hospitals, high-resource settings, or digitally connected families may perform poorly for rural households, migrant families, low-income women, ethnic minorities, families with limited literacy, or parents with low digital access. Cultural preferences in music, childbirth experience, family structure, and communication styles may also affect the acceptability of arts-based support. Before scaling up, AI-assisted recommendations should therefore be evaluated for fairness, subgroup performance, cultural appropriateness, and unintended exclusion.

### 4.3 Equity, cost, workflow, and implementation barriers

The public health value of AI-embedded arts-based care depends on whether it improves access rather than creating a new layer of technological inequality. Families with high needs, including low-income families, rural households, migrant populations, high-risk pregnancies, families of preterm infants, and parents with limited digital literacy, may be least able to use platform-based services (48, 49). For this reason, digital support should be accompanied by offline services, in-person follow-up, community health worker support, and low-cost arts-based resources.

Implementation also requires attention to cost and workflow. Even low-risk arts-based interventions require staff time, training, coordination, referral pathways, and quality assurance. AI-enabled platforms may add additional costs related to procurement, maintenance, cybersecurity, data governance, system integration, and staff training. Without institutional support, reimbursement mechanisms, and clear responsibility allocation, such frameworks may remain limited to short-term pilot projects rather than becoming sustainable public health services.

A feasible implementation strategy should start with low-risk and low-cost settings. Examples include music relaxation in prenatal education, documentation of music preferences in birth planning, brief art-based emotional support after postpartum screening, supervised parent–infant voice interaction in NICUs, and community-based parent–infant singing activities. Moderate- and high-risk cases should be connected to professional assessment and referral. In this way, the framework can be tested gradually without overstating the maturity or clinical effectiveness of AI-embedded arts-based care.

## 5 Limitations and future research

This perspective has several limitations. First, the proposed AI-embedded integrative arts-based care framework is a conceptual framework rather than a clinically validated intervention model. The purpose of this article is to synthesize existing evidence, identify integration gaps, and propose a public health-oriented pathway for future development. It does not provide original clinical data, effectiveness estimates, or implementation outcomes. Therefore, the framework should be understood as a theoretically informed proposal that requires empirical testing before being translated into routine maternal and infant health services.

Second, the evidence reviewed in this article mainly concerns selected arts-based and non-pharmacological interventions in specific perinatal and neonatal settings. Music listening, music therapy, artistic expression, doula support, parent–infant singing, and parental voice interaction have been examined in relation to pregnancy-related anxiety, labor pain and anxiety, cesarean birth experience, postpartum emotional distress, neonatal comfort, and family participation. However, these studies vary in intervention modality, timing, frequency, provider, population, outcome measurement, and follow-up duration. Future research should therefore develop clearer intervention protocols, use validated outcome measures, extend follow-up periods, and examine effects across different clinical, cultural, and socioeconomic contexts.

Third, the AI-related components of the framework are based mainly on indirect evidence from digital health, perinatal mental health screening, machine learning, and clinical decision support. Existing studies suggest that digital and AI-assisted tools may support screening, risk prediction, follow-up, and information linkage in maternal and infant health contexts (33, 34). In this Perspective, these functions are extended conceptually to arts-based care coordination. Their specific feasibility, safety, acceptability, explainability, workflow compatibility, and governance requirements in AI-embedded arts-based care remain to be tested.

Finally, future research should move gradually from conceptual development to empirical evaluation. Initial work may begin with low-risk pilot projects, such as music-based relaxation in prenatal education, preference documentation in birth planning, postpartum arts-based emotional support, supervised parent–infant voice interaction in NICUs, and community-based parent–infant singing activities. Subsequent studies should include feasibility research, implementation studies, prospective cohort designs, pragmatic trials, cost-effectiveness analyses, and equity evaluations. Particular attention should be paid to low-income households, rural families, migrant populations, high-risk pregnancies, and families of preterm infants, so that AI-embedded arts-based care expands rather than restricts access to supportive maternal and infant health services.

## 6 Conclusion

This Perspective proposes an AI-embedded integrative arts-based care framework for maternal and infant wellbeing. Its contribution lies in reframing arts-based care from isolated non-pharmacological activities into a continuous, personalized, family-participatory, and public-health-oriented pathway across pregnancy, labor, cesarean birth, postpartum recovery, NICU care, and home and community follow-up. The framework also clarifies that AI should function only as supportive coordination infrastructure for risk identification, needs profiling, personalized matching, dynamic monitoring, adaptive feedback, referral and linkage, and continuity under professional supervision. It cannot diagnose, replace professional care, or manage high-risk situations independently. If implemented with clinical safety, explainability, data protection, cybersecurity, algorithmic fairness, legal accountability, workflow integration, cost consideration, and equitable access, AI-embedded integrative arts-based care may offer a responsible direction for strengthening maternal and infant wellbeing within public health systems.

## Data Availability

The original contributions presented in the study are included in the article/supplementary material, further inquiries can be directed to the corresponding author.
